# How externalities impact an evaluation of strategies to prevent antimicrobial resistance in health care organizations

**DOI:** 10.1186/s13756-017-0211-2

**Published:** 2017-06-02

**Authors:** Jenine R. Leal, John Conly, Elizabeth Ann Henderson, Braden J. Manns

**Affiliations:** 1 Infection Prevention and Control, Alberta Health Services, Calgary, Canada; 2 Department of Community Health Sciences, University of Calgary, Calgary, Canada; 3 Cumming School of Medicine, University of Calgary, Calgary, Canada; 4 Health Sciences Centre, Room G236, 3330 Hospital Drive NW, Calgary, AB T2N 4N1 Canada; 5 Departments of Medicine, University of Calgary, Calgary, Canada; 6 Departments of Microbiology, Immunology and Infectious Diseases, University of Calgary, Calgary, Canada; 7 Departments of Pathology and Laboratory Medicine, University of Calgary, Calgary, Canada; 8 Snyder Institute for Chronic Diseases, University of Calgary, Calgary, Canada; 9 O’Brien Institute for Public Health, University of Calgary, Calgary, Canada; 10 Foothills Medical Centre, AGW5, 1403 29th Street NW, Calgary, AB T2N 2T9 Canada

**Keywords:** Antimicrobial resistance, Externality, Economics, Economic evaluation, Regulation, Taxes, Permits

## Abstract

**Background:**

The rates of antimicrobial-resistant organisms (ARO) continue to increase for both hospitalized and community patients. Few resources have been allocated to reduce the spread of resistance on global, national and local levels, in part because the broader economic impact of antimicrobial resistance (i.e. the externality) is not fully considered when determining how much to invest to prevent AROs, including strategies to contain antimicrobial resistance, such as antimicrobial stewardship programs. To determine how best to measure and incorporate the impact of externalities associated with the antimicrobial resistance when making resource allocation decisions aimed to reduce antimicrobial resistance within healthcare facilities, we reviewed the literature to identify publications which 1) described the externalities of antimicrobial resistance, 2) described approaches to quantifying the externalities associated with antimicrobial resistance or 3) described macro-level policy options to consider the impact of externalities. Medline was reviewed to identify published studies up to September 2016.

**Main body:**

An *externality* is a cost or a benefit associated with one person’s activity that impacts others who did not choose to incur that cost or benefit. We did not identify a well-accepted method of accurately quantifying the externality associated with antimicrobial resistance. We did identify three main methods that have gained popularity to try to take into account the externalities of antimicrobial resistance, including regulation, charges or taxes on the use of antimicrobials, and the right to trade permits or licenses for antimicrobial use. To our knowledge, regulating use of antimicrobials is the only strategy currently being used by health care systems to reduce antimicrobial use, and thereby reduce AROs. To justify expenditures on programs that reduce AROs (i.e. to formally incorporate the impact of the negative externality of antimicrobial resistance associated with antimicrobial use), we propose an alternative approach that quantifies the externalities of antimicrobial use, combining the attributable cost of AROs with time-series analyses showing the relationship between antimicrobial utilization and incidence of AROs.

**Conclusion:**

Based on the findings of this review, we propose a methodology that healthcare organizations can use to incorporate the impact of negative externalities when making resource allocation decisions on strategies to reduce AROs.

**Electronic supplementary material:**

The online version of this article (doi:10.1186/s13756-017-0211-2) contains supplementary material, which is available to authorized users.

## Background

Rates of antimicrobial resistant organisms have increased in the past decade among hospitalized and community patients [1, 2]. In the United States, the Centers for Disease Control and Prevention has estimated that 2 million patients a year have infections due to antimicrobial resistant bacteria leading to 23,000 deaths annually [3]. One of the significant determinants of antimicrobial resistance is the selection pressure placed by the use, misuse and overuse of antimicrobials which provides a comparative advantage to the small fraction of organisms naturally resistant to the antimicrobials [4–6]. It is estimated that approximately 30% of oral antimicrobial prescriptions from ambulatory visits in the United States are inappropriately prescribed to patients [7]. Patients infected with an antimicrobial resistant organism experience increased treatment failure with subsequent use of more expensive treatment, extra investigations, longer hospital stays, longer time off work and most importantly premature death [4]. Additionally, if antimicrobials lose their effectiveness, key medical procedures could become too dangerous to perform [8].

In addition to the impact on patients, antimicrobial resistance also impacts the economy by delaying or reducing the number of individuals that can return to the workforce due to increased morbidity and early death among those with an ARO.Health care costs also increase through additional or longer treatments, and longer hospital stays, for the patient with the ARO, and the additional patients who become infected with it [5, 6]. For example, the estimated annual direct and indirect costs of antimicrobial resistance is $55 billion in the United States [3]. It is also projected that by 2050, 10 million lives a year and a cumulative USD $100 trillion of economic output are at risk due to the increase in antimicrobial resistant infections [8]. Moreover, there are costs of introducing new antimicrobials to replace old, ineffective ones and opportunity costs of committing these resources that could be used for other public health initiatives [5, 6].

Despite these significant consequences associated with antimicrobial resistance, few resources have been allocated to reduce the spread of resistance on global, national and local levels, in part because the broader economic impact of antimicrobial resistance is often not fully considered when determining how much to invest to prevent AROs, including strategies to contain antimicrobial resistance, such as antimicrobial stewardship programs [2, 9]. Estimates of costs associated with resistance have been extremely crude, limited and likely an underestimate as they have primarily focused on costs incurred by health care systems and not society, have focussed on current rather than future potential costs, and have focussed on costs in the developed rather than the developing world [4, 10]. Antimicrobial resistance has been conceptualized in health economics as a negative externality (defined below) associated with antimicrobial use.

### The economic concept of externalities and its application to antimicrobials: a condition of market failure

Markets will distribute resources efficiently when both consumers and producers consider all the effects of their actions when exchanging goods [11, 12]. In some instances the benefit or cost of production or consumption of goods to society differ from the individual private benefit or cost [11]. However, self-interested consumers and producers often only consider costs and benefits to themselves and not others [11, 12].

An *externality* is a cost or a benefit associated with one person’s activity (i.e. the production or use of a good or service) that impacts others or society who did not choose to incur that cost or benefit. For example, the influenza vaccine provides private, individual benefit to the patient receiving it by protecting that patient from acquiring the infection, but also carries external benefits with reduced transmission to other susceptible people [11]. The externality, or the difference between the individual benefit (cost) and the social benefit (cost), are not accounted for in market transactions [12].

Externalities cause inefficient allocation of resources by markets because people in the market exchange goods based on their individual costs and benefits and not on the broader social benefits and costs [11]. If a commodity or good produces positive effects to society beyond the individual supplier or consumer, the commodity or good will be under produced or utilized. To continue with the influenza example above, the external benefit of the influenza vaccine to society is usually not considered by individuals choosing whether to get vaccinated, and this may result in a socially less optimal level of influenza vaccine use and an overall welfare loss to society [11].

Alternatively, if producers or consumers of commodities that result in negative effects beyond the individual to society do not bear the full costs of such effects, too much of the commodity will be produced [12]. Antimicrobial use results in both positive and negative spillover effects. The positive effect to others and society associated with antimicrobial use is related to the decreased morbidity from sub-clinical infections that are treated accidently and decreased transmission of microbes to other susceptible patients [4, 13]. Antimicrobial resistance is a negative externality from the consumption of antimicrobials to produce health [4, 13]. Antimicrobial resistance has effects that are unlikely to be felt directly by either the patient using or physician providing the antimicrobial, but has adverse effects on future patients who may require antimicrobials and on health care systems more broadly thus impacting the overall welfare of society [13, 14]. Resistance imposes large costs on society in the form of increased hospitalizations, higher mortality rates, decreased effectiveness of antimicrobials for current and future generations, and the use of resources into the development of new more powerful antimicrobials which could be used for other medical needs [15].

Health economists have begun to consider possible policy responses to tackle antimicrobial resistance as a result of the use, misuse and overuse of antimicrobials [4, 6, 13, 16, 17]. The objective of this paper was to provide an overview of externalities in the context of antimicrobial use and to explore the optimal way to measure and formally consider the positive or negative societal effects (i.e. positive or negative externalities, respectively) associated with antimicrobial use when making resource allocation decisions aimed to reduce antimicrobial resistance within healthcare facilities.

## Methods

We conducted a review of the literature to identify studies which described: 1) externalities of antimicrobial use, 2) approaches to quantifying the externalities associated with antimicrobial resistance or 3) policy options both at a macro and a micro level that incorporate the externalities associated with antimicrobial resistance. We hypothesized that with collation of the findings from the literature review we could propose methods to measure and incorporate the impact of externalities of antimicrobial use when evaluating strategies to reduce antimicrobial resistance in healthcare organizations or facilities.

### Inclusion/exclusion criteria

All relevant literature describing externalities of antimicrobial use, ways to measure externalities from antimicrobial use within healthcare facilities, or policy options which consider these externalities were included. Articles which evaluated externalities of antimicrobial use among animals or described policy options to reduce antimicrobial use among animals were excluded.

### Types of publications

Reviews, case report/series, narrative reviews, chapter textbooks, editorials, commentaries, and content analysis papers were included to ensure a comprehensive collection of literature addressing externalities associated with antimicrobial resistance. Studies presenting economic models or infectious diseases transmission models linked with economic models were included.

### Search method

On September 27, 2016 a search for relevant articles using the online databases Medline (OVID) and EMBASE was performed. Only English articles were eligible for inclusion. The initial search of online databases included all languages and was not limited by publication year or geography; however only English articles were eligible for inclusion. The first Boolean searches were conducted using the term “or” to explode (search by subject heading) and map (search by keyword) the following MeSH headings and keywords “Anti-Bacterial Agents”, “Anti-Infective Agents”, “Drug Utilization”, and “antimicrobial”. The second Boolean search was done using the term “or” to explode and map “Taxes”, “Fees and Charges”, and “Regulation” and “permits.” The third Boolean search was done using the term “or” to explode and map “Economics” and “externality.” The first and second Boolean searches were combined with “and” to identify articles related to antimicrobial policy options. The first and third Boolean searches were combined with “and” to identify articles related to antimicrobial use, resistance and externalities. All searches were combined in a single search strategy (Additional File 1). All potential titles and abstracts were extracted from Medline (OVID) and EMBASE and managed using Endnote X7.7 (Thomson Reuters, 1988–2016).

## Results

**Fig. 1:**
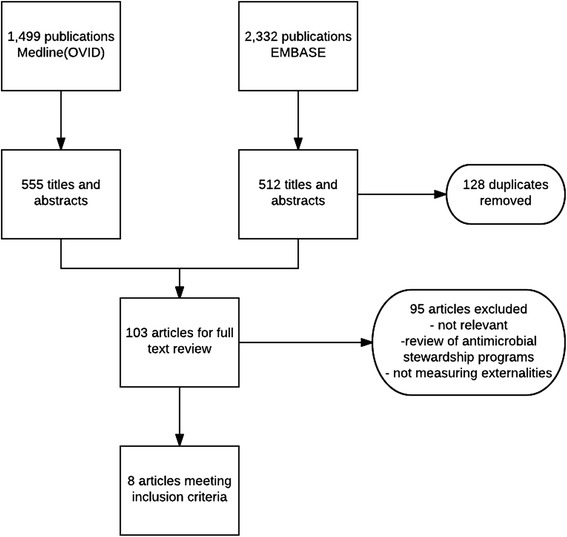
Flow diagram of article selection

The initial review of titles and abstracts resulted in 555 articles from Medline (OVID) and 512 articles from EMBASE. Once duplicates were removed a total of 939 articles remained for further review. Of these, 103 articles were extracted for full text review to assess their eligibility (Fig. 1). Only four articles presented models evaluating the impact of antimicrobial use on antimicrobial resistance in an attempt to estimate the magnitude of the negative externality associated with antimicrobial utilization [5, 18–20]. Four articles discussed macro policies of regulations, taxes/charges and permits to contain antimicrobial resistance [13, 17, 21, 22]. These articles are summarized in Table 1 and discussed after a brief overview of the methods used to measure the externalities of antimicrobial use.

**Table 1:** Study characteristics

Author	Year	Study type	Outcome measured	Results ($US 2016)
Phelps [20]	1989	Economic model	Costs and benefits associated with antimicrobial use in the United States	Unrecognized social cost annually in the US ranging from $145 million to $14.5 billion
Rudholm [21]	2002	Economic Models	Antimicrobial resistance at global level and to design a tax/subsidy system	No empirical analysis used to simulate models
Elbasha [19]	2003	Economic model	Excess burden to society from extra courses of amoxicillin and amoxicillin/clavulanate use, based on Phelps model	Excess burden associated with 40 million prescriptions was $345 million
Smith et al. [17]	2006	Economic model	Evaluating regulation, taxation, tradeable permits on reducing antimicrobial use and level of MRSA	Taxation least effective, free tradeable permit most effective
Kaier and Frank [18]	2010	Economic model	Cost of resistance and of antimicrobial consumption (negative externalities) and use of alcohol based hand rub for hand disinfection (positive externality) on the incidence of hospital-acquired MRSA	Negative externality associated with 2nd generation cephalosporins, 3rd generation cephalosporins, fluoroquinolones and lincosamines ranged $6–17 per DDD
Kaier and Volkswirt [5]	2012	Economic model	Cost of resistance and of antimicrobial consumption (negative externalities) and use of alcohol based hand rub for hand disinfection (positive externality) on the incidence of hospital-acquired MRSA, CDAD, ESBL	Negative externality associated with 3rd gen cephalosporins ($159 per DDD) and fluoroquinolones ($112 per DDD).
Smith & Coast [22]	1998	Description	Conceptual and practical issues of tradeable permits	Identified 7 factors to consider
Coast et al. [13]	1998	Description	Resistance as an externality and policy options (e.g. regulation, taxation, tradeable permits) for dealing with antimicrobial resistance	Strengths and limitations of policy options

*MRSA* Methicillin resistant *Staphylococcus aureus, CDAD Clostridium difficile-*associated diarrhea, *ESBL* extended spectrum beta-lactamase, *DDD* defined daily dose

### Ways to measure externalities of antimicrobial use

In terms of antimicrobial use, all the costs and benefits need to be considered, including those due to externalities to inform decision makers on strategies to contain or have an impact on antimicrobial resistance [4]. The net benefit from antimicrobial use will depend on the relative sizes of its negative and positive externalities, direct benefit to the patient, treatment and administrative costs, side effects to the patient, cost associated with difficulty in diagnosing an infection because of the use of antimicrobials, the amount of antimicrobial consumed, and other factors (e.g. natural level of resistance in the population, resistance from previous time periods, population mobility and density) [4, 13]. There are few robust and complete estimates on the cost impact of antimicrobial resistance and they have been largely ignored in economic evaluations to date [2, 4, 14, 16].

The cost impact of antimicrobial resistance may start with determining the attributable cost of AROs by comparing costs between groups of patients with and without the ARO or by creating prediction models to describe determinants of the variation in costs between patients with and without the ARO [23]. Different regression models have been used to determine attributable costs, while controlling for confounders and possible effect modifiers. These have included: normal distribution-based models (e.g. ordinary least squares), methods following transformation of data, generalized linear models, two or multi-part models, and Cox proportional hazards model [23–28]. Recently, there has been more interest in methods based on the propensity score to reduce or eliminate bias and the effects of confounding when using observational data instead of regression adjustment [29]. Propensity score matching involves matching on the propensity score for a patient which is the probability of having an ARO conditional on the patient’s observed baseline characteristics rather than inefficiently matching on multiple individual risk factors correlated with both costs and the ARO [30]. A recent study comparing three estimation strategies to determine the attributable cost of healthcare-associated Methicillin-resistant *Staphylococcus aureus* (MRSA) infections found that propensity score matching reduced the time-dependent bias substantially by matching on the timing of infection [31]. Failure to account for the time-varying nature of healthcare-associated infections results in an overestimation of the impact of healthcare-associated infections on length of stay and costs [31, 32]. Currently, there is no guidance on which approach is preferred, however determining the attributable cost of AROs is crucial in determining whether resources may be allocated to strategies to reduce antimicrobial resistance [23].

Charles E. Phelps [20] created an economic model to determine the potential magnitude of the unrecognized social cost or externality associated with antimicrobial use in the United States within 1 year by using evidence available in the literature. The model included the costs and benefits of using an antimicrobial and incorporated the possibility of enhanced resistance as the overall rate of antimicrobial use increased. For 1 year of resistance, the unrecognized social cost accruing annually in the United States ranged from $145 million to $14.5 billion, based on 150 million prescriptions per year [20]. If resistance from current antimicrobial use lasted five years, then the costs would be 4.7 times greater, ranging from $0.68 billion to $68 billion (2016 $US) [20].

Elamin H. Elbasha [19] created a simple model of antimicrobial use and resistance based on the model by Phelps [20] but focused on the size of the excess burden to society from resistance compared to the benefit resulting from the extra courses prescribed. It also accounted for infections with organisms other than bacteria that can cause different types of adverse health outcomes. Finally, it also considered the behaviours of various parties (e.g. drug companies) and their role in controlling the development and spread of resistance. He focused on estimating the excess burden of outpatient prescriptions for amoxicillin and amoxicillin/clavulanate in the United States in 1996 [19]. The excess burden associated with 40 million prescriptions for amoxicillin and amoxicillin/clavulanate was $345 million (2016 $US) and it was found to be most sensitive to changes in the estimates of price and changes in demand [19].

Kaier and Frank’s [18] study determined the negative externalities, which he equated to the cost of resistance and of antimicrobial consumption and positive externalities from the use of alcohol based hand rub (ABHR) for hand disinfection. A simple model was created which combined results from cost-of-illness studies and multivariate time-series analyses, which determined the impact of antimicrobial consumption and ABHR use on the incidence of hospital-acquired infections caused by MRSA. In this approach, the externality was defined as the cost of resistance that is caused by use of one defined daily dose (DDD) of a selected antimicrobial. Their results demonstrated the external cost of antimicrobial consumption resulting from promoting resistance and found that each DDD of second-generation cephalosporins, third generation cephalosporins, fluoroquinolones and lincosamides was associated with an externality of about $6–16.7 ($US 2016) [18].

In this study, only MRSA infections were considered and thus would underestimate the externality because antimicrobial use also affects the emergence and spread of other multidrug resistant bacteria; it did not include behavioural relationships as shown by Elbasha and Phelps and only focused on the cost of resistance due to treatment without taking into account benefits of antibacterial use [18–20].

In a subsequent analysis by Kaier and Volkswirt [5], a similar economic model was used to evaluate the external effects of using broad-spectrum antimicrobials and ABHR with respect to the emergence of antimicrobial resistance. This study also included cost-of-illness studies of nosocomial MRSA, *Clostridium difficile*-associated diarrhea (CDAD), Extended Spectrum Beta-lactamases (ESBL) and time-series analysis evaluating the relationship between these infections with antimicrobial use, ABHR, and other factors such as bed occupancy rates, turnover intervals, and average length of hospital stay on the spread of MRSA, ESBL and CDAD. They found substantial negative externality associated with third-generation cephalosporins and fluoroquinolones (2016, US$159 and $112, respectively) because their use was shown to influence the incidences of MRSA, CDAD and ESBL [5].

### Strategies to take into account the externalities of antimicrobial resistance

From the literature review, we identified three macro level strategies aimed at containing antimicrobial resistance which would assist patients, clinicians and the health care system to take into account the negative externality of antimicrobial resistance through some form of intervention [4]. Three methods to account for the negative externality from the consumption of antimicrobials among patients and prescribers are regulation, charges or taxes on the use of antimicrobials, and the right to trade permits or licenses [4, 13].

Regulation could include limiting the use of antimicrobials for particular types of patients by regulating the medical conditions for which antimicrobials would be appropriate and allowable [13]. This could be enforced through formulary restrictions in hospital settings whereby the hospital pharmacists can override the decisions of prescribing practitioners, or it could limit the total supply of antimicrobials available to physicians for prescription to their patients by regulating the total quantity of antimicrobials that any single physician can prescribe [13, 33].

A second approach that was identified was for governments or healthcare organizations to impose a user charge or tax to get the optimal level of production and consumption of antimicrobials [4, 13]. The key to imposing a charge or tax so that those consuming or producing antimicrobials can bear the full burden of the cost is that the tax or charge should equate the externality associated with antimicrobial use [4, 13, 17]. This would reduce the use of antimicrobials and the rate at which resistance develops [34]. Furthermore, the revenue raised could be used to subsidize research and development into new antimicrobials [6, 34].

A third approach involves combining regulation which is relatively easy to administer with the flexibility and efficiency of a tax or charge through tradeable permits [4, 17]. In this approach, the total quantity of antimicrobials is limited through regulation. Each prescriber would have permits to prescribe up to a designated level of different types of antimicrobials during a particular time but they would be capped at the total amount of prescriptions they could prescribe [13, 17]. Beyond the initial entitlement in their permit, prescribers or physicians could purchase additional permits for particular types of antimicrobials from other physicians who do not readily use antimicrobials in their practice [13]. Those prescribers with lower marginal costs of reducing their limit of antimicrobials can sell their permit allocation for a profit to those with higher marginal costs [17]. For those purchasing the permits, the choice to purchase the additional permits is based on when the benefit associated with having additional antimicrobial prescriptions is greater than the benefit associated with having other types of prescriptions [13].

Of the four articles identified that discussed the three macro policies above, Smith et al. [17] was the only one that evaluated the impact of antimicrobial resistance, specifically of MRSA, and the three macro policies to contain antimicrobial resistance, not only on the healthcare sector but also on larger economic indicators such as national income, labour supply, gross domestic product and economic growth [35]. A computable general equilibrium model was used to evaluate the effects of economic and policy shocks to the economy as a whole. When comparing the three policy options, regulation (with an assumed decrease of antimicrobial use by 10%) would improve household income, tax revenue, real gross domestic product (GDP) and total national savings between 0.014 and 0.101% [17]. A tax rate of 10% on the prescription of antimicrobials would lead to an approximate reduction in the level of MRSA of just under 10% [17]. This also resulted in decreased unemployment and government expenditure (0.431, 0.075% respectively) and increased real GDP (0.039%) [17]. A free tradeable permit policy where the prescription allowance decreased by 10% resulted in increased household income by 0.015%, unemployment declined by 1% and real GDP increased by 0.08% [17]. Overall, a policy of taxation appeared to be the least effective, where the use of tradeable permits was the most effective in reducing the prescription of antimicrobials and the level of antimicrobial resistance [17].

While we are unaware of any countries currently using tradable permits, Smith & Coast [22] described the use of tradeable permits, including the conceptual and practical issues which should be considered before operationalizing such a system. Seven factors were identified that would need to be addressed in the development of this market: system objective (e.g. cost-effective reductions in antimicrobial prescribing); means to enable trade (i.e. a budget to allow trade of prescriptions for antimicrobials); initial allocation of permits to prescribers; differentiation of permits by locality, type of antimicrobial and across time; and how the system might be enforced [22].

### Limitations of existing strategies to take into account externalities associated with antimicrobial use

As noted, three macro policies (regulations, taxes, tradeable permits) have been described to contain antimicrobial resistance which aim to account for the negative externality of antimicrobial resistance through some form of government intervention [4]. They entail regulations on prescribers or providing incentives for users and prescribers to take into account the possible external costs to society from using antimicrobials.

Although regulations would be easy to introduce and administer there are some significant limitations. Firstly, the issue of heterogeneity among patients makes it difficult to account for all patients’ illnesses and circumstances [4, 13]. Secondly, there is the issue of physician compliance with the rules about who should or should not receive the antimicrobial treatment [13]. Ensuring compliance can entail heavy transaction costs, may be perceived to conflict with physicians’ clinical freedom and a uniform regulatory policy may impose higher costs in one location over another because antimicrobial use varies from place to place, as does the burden of infectious diseases and outcomes [13]. A simpler approach would be to create clinical guidelines or antimicrobial stewardship principles instead of regulations, which already occurs in various healthcare organizations [13].

The difficulties with introducing a tax on antimicrobial use are not only the complexity of administering it but also in determining what the marginal external cost is of antimicrobial resistance to determine what tax or charge would be appropriate [17]. Given some of the challenges noted above about the nature of the negative externality of antimicrobial resistance, this may be difficult and impractical. One approach considered is to use the consumption of antimicrobials as a proxy for generating antimicrobial resistance and thus relating the tax or charge to the level of consumption [5, 18]. This information is readily available in most hospitals and health care systems and would be easy to quantify [4]. However, there is limited time series data on both antimicrobial use and antimicrobial resistance making it difficult to estimate the dose-response relationship between antimicrobial use and resistance [6]. It is also unclear who will bear the costs of the negative externality by paying the taxes or charges. The possibilities are having the patient who consumes the antimicrobial, or the clinician who prescribes, or the third party payer that pays for necessary care or the firm that produces it [13]. When a third party payer covers the full cost of the antimicrobial, as is the case in Canadian hospitals, it is unlikely to create incentives for physicians to prescribe less or for the patient to consume less [13]. Having the patient pay continues to pose problems since the demand for antimicrobial use may be inelastic, meaning that as the price of the antimicrobial increases, the quantity of antimicrobial consumed may not fall significantly [13]. Another alternative is to apply a charge to physicians who operate within a defined drugs budget [13]. A tax or charge could be applied to antimicrobials specifically that would impact the overall drug budget, which may provide an incentive for physicians to reduce prescriptions of antimicrobials in relation to prescriptions of other drugs, to select substitute therapies for minor self-limiting conditions, and to consider most appropriate antimicrobials for more severe conditions [13]. This could work well for physicians in primary care or community care where physicians may have direct oversight or knowledge of these budgets, but may not provide the same incentive to reduce prescribing among physicians in a hospital environment where budgets are not managed by individual physicians.

The difficulty in implementing tradeable permits as the third policy option lies with the required system of strict regulation and its challenges of enforcement and policing to ensure compliance by prescribers [13, 17]. This strategy may be difficult to manage and would come with the perception among physicians/prescribers of added barriers [13, 17]. Furthermore, the allocation of permits would likely be based on historical use of drugs and antimicrobials, which is not as equitable as using a formula-based approach that considers the variation in the patient population that the physician serves [13]. Finally, this approach may not directly result in research and development of new antimicrobials since the tradeable permits and associated profits would be among prescribers [13].

### Limitation in existing methods for measuring externalities associated with antimicrobial use

While our literature review identified three ways to account for the externality associated with antimicrobial use through regulation, taxes, or tradable permits, the challenge more broadly is that empirical evidence on the size of externalities associated with antimicrobial use is limited [11]. Our review demonstrates the paucity of literature on methods to quantify the magnitude of externalities associated with antimicrobial use, particularly in hospitalized settings which may partly be explained by our search strategy but more likely due to limited study on the concept of externalities associated with antimicrobial use. Furthermore, many factors that could influence cost estimates associated with antimicrobial resistance are not considered in existing studies, including rising prevalence of antimicrobial resistance, consequences of the unavailability of effective antimicrobials where they are most commonly used, and the effect of antimicrobial resistance on broader economic indicators such as national income, labour supply and economic growth [35]. However, the studies by Kaier et al. present models which may be adopted and used using local healthcare data as an initial first step [5, 18].

In addition to measurement problems, there are additional aspects of the negative externality of antimicrobial consumption that may contribute to the underreporting of measurement and valuation of costs associated with antimicrobial resistance and policies aimed at containing it [16]. They include the intergenerational, interregional nature of antimicrobial resistance, and the uncertainty associated with antimicrobial resistance [13, 16]. The intergenerational nature of the problem arises because the effects of antimicrobial resistance impacts both current and future generations [13]. In an evaluation of policies to reduce antimicrobial resistance, currents costs of reducing antimicrobial consumption need to be weighed against the future costs of not reducing consumption [4, 13]. The problem with this is that future generations have no voice in policy decisions made in the present [13]. Society’s perception of the costs associated with antimicrobial resistance may be diminished because the resistance from the use of antimicrobials is expected to occur in the future [14, 16].

The interregional problem of antimicrobial resistance arises because antimicrobial resistance extends and varies across different locations and can change rapidly in a single location [13]. One region or country may be the source and victim of increased antimicrobial resistance and the transfers between regions and countries may be unequal in quantity [13]. Policies to reduce antimicrobial use in one region or country may be ineffective because the spread of antimicrobial resistant organisms between regions or countries may have a greater impact than the policy [13].

There are many areas of uncertainty surrounding antimicrobial resistance, including the costs of resistance, the mechanisms organisms use to develop resistance, the relationship and timing between the development of resistance and the use of a particular antimicrobial, the potential for developing a new antimicrobial to which there is not yet resistant organisms, and the potential for reducing resistance that has already developed by reducing the use of the antimicrobial [4, 13]. All of these uncertainties need to be considered when determining the impact of the negative externality associated with antimicrobial resistance and developing strategies to contain antimicrobial resistance.

One final problem associated with assessing the value of these different policy responses is identifying their influence on resistance. Literature on educational interventions, restrictions, regulations intended to reduce prescribing is aimed at reducing emergence of resistance at the community level but the impacts tend to be measured in terms of changes in antimicrobial prescribing as a proxy for impact on resistance and thus health [16, 33, 36]. However, it is the indirect impact on individuals not directly targeted by the policy which will result in the greatest benefit and increases the value of the policy in an economic evaluation [16].

### Proposed methods to incorporate externalities associated with antimicrobial use by healthcare organizations

Although various healthcare organizations have introduced policies and antimicrobial stewardship programs to regulate the use of antimicrobials to promote the appropriate use of antimicrobial agents, a clear strategy to analyse the effectiveness and costs of these policies, and to take into account the negative externality associated with antimicrobial use, have not been formally evaluated. When determining whether to invest in a new strategy all relevant changes to costs and health benefits achieved, including the externalities associated with antimicrobial consumption should be quantified and compared to understand if the intervention offers value for money [36]. As the costs related to the reduction or delayed emergence of resistance is not currently formally undertaken in many healthcare organizations, further work is needed to quantify the externalities of antimicrobial use within hospitals particularly that associated with antimicrobial resistance.

With the movement towards using administrative and electronic healthcare data to measure a number of healthcare indictors and resource utilization it is possible in many jurisdictions to quantify key factors that are required to estimate the negative externality associated with antimicrobial use. Data on these key factors (discussed in Table 2 below), can inform a simple economic model like that developed by Kaier et al. which describes the impact of antimicrobial use on the development of antimicrobial resistance [5, 18]. The cost associated with antimicrobial resistance can then be quantified using existing costing systems.

**Table 2:** Proposed method that could be undertaken by healthcare organizations to incorporate externalities associated with antimicrobial use

Measurements required	Description	Data sources
Strategy description and costs	Describe the strategy to contain antimicrobial resistance by reducing the use of antimicrobials in hospitals. Describe all costs associated with implementing and running the program for a minimum of 2 years.	Existing Antimicrobial Stewardship programs, Pharmacy Services, Financial departments
Baseline rate of antimicrobial resistant organism	Rates of hospital-acquired, healthcare associated or community-acquired infections due to antimicrobial resistant organisms in the healthcare facility	Infection Prevention and Control Laboratory microbiology data linked to Discharge Abstract Databases
Strategy effectiveness	Policy effectiveness would be measured by the change in the dose-response relationship (elasticity) between antimicrobial consumption and the emergence of resistance using multivariate time series analyses accounting for different factors related to transmission of the antimicrobial resistant organism. The model coefficients would demonstrate that temporal increases/decreases in the volume of antimicrobial use are followed by temporal increases/decreases in the incidence of hospital acquired antimicrobial resistant organism.	Infection Prevention and Control Laboratory microbiology linked to Discharge Abstract Databases Antimicrobial utilization from patient management systems or pharmacy prescribing systems
Incremental cost of Antimicrobial resistant infections	The incremental costs of infections with antimicrobial resistant organisms is required whereby systematic differences between patients with and without the infection are accounted for. These differences can be accounted for by a particular epidemiologic study design (i.e. matching) or through statistical modeling techniques. There is a paucity of studies currently that appropriately evaluate the true incremental costs of these infections and which consider the time-varying nature of healthcare-associated infections^a^	Hospital financial departments with micro-costing data on costs/charges of patients with and without infections with an antimicrobial resistant organism.
Economic evaluation of the strategy	A simple decision analytic model to compare the costs of the policy with the costs saved through reducing antimicrobial resistant organisms (the negative externality). It would evaluate the change in the dose-response relationship between antimicrobial use and antimicrobial resistance as a result of the policy. The time horizon would be a minimum of 2 years to observe changes in antimicrobial resistance rates as a result of reduced prescribing.	

^a^This concept is further discussed in the section “Ways to Measure Externalities of Antimicrobial Use”

The approach by Kaier et al. focuses on in-hospital antimicrobial treatment and the use of alcohol based hand rub on the effect of hospital-acquired infections caused by antimicrobial resistant organisms [5, 18]. The results reflect the external costs of antimicrobial consumption which promotes resistance and could be presented as the externality cost associated with one defined daily dose of the antimicrobial. The results may be used as a rough estimate of the indirect costs of in-hospital antimicrobial use which can be applied in economic evaluations considering the costs and consequences of strategies to contain antimicrobial resistance (Table 2).

Although a simple, static model, this may be an appropriate first step using available data and published economic models to estimate the negative externality associated with antimicrobial use in healthcare facilities. Future work may involve the use of mathematical models consisting of compartments (e.g. Susceptible-Infected-Susceptible model) and dynamic changes in the uninfected population and those infected with sensitive or resistant strains [37–39]. It would model the development of antimicrobial resistance (i.e. reduced sensitivity or effectiveness over time) with the use of particular antimicrobials. Although dynamic models may be more appropriate for evaluating the impact of antimicrobial use on the emergence and transmission of antimicrobial resistant organisms, the simple approach proposed above can be the first step to encourage healthcare organizations to consider externalities associated with antimicrobial use which may be incorporated into studies evaluating the cost-effectiveness of different prevention strategies for antimicrobial resistance.

## Conclusion

Loss of antimicrobial effectiveness as a result of antimicrobial consumption can be considered a negative externality. Since individual patients and prescribers do not bear the impact of this negative externality, this results in the overconsumption of antimicrobials. Strategies to take into account the negative externality associated with antimicrobial resistance have centred on regulation, taxation and tradeable permits. The latter two are economic approaches that are more effective at reducing antimicrobial consumption than regulation alone as they address some of the missing economic incentives among users and prescribers; however they have not been used to reduce antimicrobial use in practice. Understanding the magnitude of the externality may inform the level of involvement by health care systems to take into account the external effects of antimicrobial use. The challenge, however is that the empirical evidence on the magnitude of the externalities is limited. It is critical to consider all costs and benefits of antimicrobial use, including those due to externalities to inform decision makers on strategies to contain or have an impact of antimicrobial resistance. We propose a simple model as a first step to estimate the magnitude of the negative externality associated with antimicrobial use in healthcare facilities that may be useful in economic evaluations of strategies to reduce antimicrobial resistance.

## Additional file

Additional file 1: Medline (OVID)/Embase Search Strategy. Table with detailed online database search strategy. (DOCX 13 kb)

## Abbreviations

ABHR: Alcohol-based hand rub
ARO: Antimicrobial resistant organism
CDAD: *Clostridium difficile-*associated diarrhea
DDD: Defined daily dose
DWL: Deadweight loss
ESBL: Extended spectrum beta-lactamase
GDP: Gross domestic product
MRSA: Methicillin resistant *Staphylococcus aureus*
