# Supplementary material for: How externalities impact an evaluation of strategies to prevent antimicrobial resistance in health care organizations
Source: Antimicrob Resist Infect Control. 2017 Jun 2;6:53. doi: 10.1186/s13756-017-0211-2 (PMC5457558; doi:10.1186/s13756-017-0211-2)
Supplement: Additional file 1: — Medline (OVID)/Embase Search Strategy. Table with detailed online database search strategy. (DOCX 13 kb) [file 13756_2017_211_MOESM1_ESM.docx]

**ADDITIONAL FILE 1**

Medline (OVID)/Embase Search Strategy

| **Medline Search Terms 27 September 2016** |  |
| --- | --- |
| 1. Anti-Bacterial Agents/ad, ae, an, ec, ut [Administration & Dosage, Adverse Effects, Analysis, Economics, Utilization] |  |
| 2. antimicrobial.mp. |  |
| 3. antimicrobial.mp. |  |
| 4. exp Anti-Infective Agents/ or antibacterial.mp. |  |
| 5. (anti?microbial or anti?bacterial or anti?infect*).mp. [mp=title, abstract, original title, name of substance word, subject heading word, keyword heading word, protocol supplementary concept word, rare disease supplementary concept word, unique identifier] |  |
| 6. exp Drug Utilization/ec, mt, og, sn, td [Economics, Methods, Organization & Administration, Statistics & Numerical Data, Trends] |  |
| 7. (drug adj2 utili?ation).mp. [mp=title, abstract, original title, name of substance word, subject heading word, keyword heading word, protocol supplementary concept word, rare disease supplementary concept word, unique identifier] |  |
| 8. exp Taxes/ |  |
| 9. tax*.mp. |  |
| 10. exp "Fees and Charges"/ |  |
| 11. (fee* or charge* or levy or levies).mp. [mp=title, abstract, original title, name of substance word, subject heading word, keyword heading word, protocol supplementary concept word, rare disease supplementary concept word, unique identifier] |  |
| 12. exp Licensure/ |  |
| 13. licensure.mp. or exp Licensure, Medical/ or exp Licensure, Hospital/ or exp Licensure, Pharmacy/ |  |
| 14. permit.mp. |  |
| 15. regulation.mp. |  |
| 16. exp Practice Guideline/ or guideline.mp. or exp Guideline/ |  |
| 17. principle.mp. |  |
| 18. exp Economics/ |  |
| 19. (externality or consequence).mp. [mp=title, abstract, original title, name of substance word, subject heading word, keyword heading word, protocol supplementary concept word, rare disease supplementary concept word, unique identifier] |  |
| 20. (antimicrobial adj2 resistance).mp. [mp=title, abstract, original title, name of substance word, subject heading word, keyword heading word, protocol supplementary concept word, rare disease supplementary concept word, unique identifier] |  |
| 21. (anti?microbial adj2 resistan*).mp. [mp=title, abstract, original title, name of substance word, subject heading word, keyword heading word, protocol supplementary concept word, rare disease supplementary concept word, unique identifier] |  |
| 22. (anti?bacterial adj2 resistan*).mp. [mp=title, abstract, original title, name of substance word, subject heading word, keyword heading word, protocol supplementary concept word, rare disease supplementary concept word, unique identifier] |  |
| 23. (anti?infect* adj2 resistan*).mp. [mp=title, abstract, original title, name of substance word, subject heading word, keyword heading word, protocol supplementary concept word, rare disease supplementary concept word, unique identifier] |  |
| 24. 1 or 2 or 3 or 4 or 5 | Antimicrobials |
| 25. 6 or 7 | Drug Use |
| 26. 8 or 9 or 10 or 11 or 12 or 13 or 14 or 15 or 16 or 17 | Policy options |
| 27. 18 or 19 | Externality |
| 28. 20 or 21 or 22 or 23 | Antimicrobial Resistance |
| 29. 24 and 25 | Antimicrobial + Drug Use |
| 30. 26 and 29 | Policy Options + Antimicrobial + Drug Use |
| 31. 27 and 28 | Externality + Antimicrobial Resistance |
| 32. 30 or 31 | Either (Policies on Antimicrobial Use) OR (Externality of Antimicrobial Resistance) |
